# Tackling Africa's chronic disease burden: from the local to the global

**DOI:** 10.1186/1744-8603-6-5

**Published:** 2010-04-19

**Authors:** Ama de-Graft Aikins, Nigel Unwin, Charles Agyemang, Pascale Allotey, Catherine Campbell, Daniel Arhinful

**Affiliations:** 1 Department of Social and Developmental Psychology, Faculty of Politics, Psychology, Sociology and International Studies, University of Cambridge, Cambridge, UK; 2 Institute of Health and Society, University of Newcastle, Newcastle, UK; 3 Department of Public Health, Academic Medical Centre, University of Amsterdam, Amsterdam, the Netherlands; 4 School of Medicine and Health Sciences, Monash University, Kuala Lumpur, Malaysia; 5 Institute of Social Psychology, London School of Economics and Political Science, London, UK; 6 Noguchi Memorial Institute for Medical Research, University of Ghana, Accra, Ghana

## Abstract

Africa faces a double burden of infectious and chronic diseases. While infectious diseases still account for at least 69% of deaths on the continent, age specific mortality rates from chronic diseases as a whole are actually higher in sub Saharan Africa than in virtually all other regions of the world, in both men and women. Over the next ten years the continent is projected to experience the largest increase in death rates from cardiovascular disease, cancer, respiratory disease and diabetes. African health systems are weak and national investments in healthcare training and service delivery continue to prioritise infectious and parasitic diseases. There is a strong consensus that Africa faces significant challenges in chronic disease research, practice and policy. This editorial reviews eight original papers submitted to a Globalization and Health special issue themed: "Africa's chronic disease burden: local and global perspectives". The papers offer new empirical evidence and comprehensive reviews on diabetes in Tanzania, sickle cell disease in Nigeria, chronic mental illness in rural Ghana, HIV/AIDS care-giving among children in Kenya and chronic disease interventions in Ghana and Cameroon. Regional and international reviews are offered on cardiovascular risk in Africa, comorbidity between infectious and chronic diseases and cardiovascular disease, diabetes and established risk factors among populations of sub-Saharan African descent in Europe. We discuss insights from these papers within the contexts of medical, psychological, community and policy dimensions of chronic disease. There is an urgent need for primary and secondary interventions and for African health policymakers and governments to prioritise the development and implementation of chronic disease policies. Two gaps need critical attention. The first gap concerns the need for multidisciplinary models of research to properly inform the design of interventions. The second gap concerns understanding the processes and political economies of policy making in sub Saharan Africa. The economic impact of chronic diseases for families, health systems and governments and the relationships between national policy making and international economic and political pressures have a huge impact on the risk of chronic diseases and the ability of countries to respond to them.

## Introduction

Africa bears a significant proportion of the global burden of chronic diseases, along with poor countries of Asia and Latin America (see appendix 1). The World Health Organisation (WHO) projects that over the next ten years the continent will experience the largest increase in death rates from cardiovascular disease, cancer, respiratory disease and diabetes [1]. Africa's chronic disease burden is attributed to multifaceted factors including increased life expectancy, changing lifestyle practices, poverty, urbanisation and globalisation [1]. Rising morbidity and mortality from chronic diseases co-exist with an even greater burden of infectious disease, which still accounts for at least 69% of deaths on the continent [2]. Many African health systems are under-funded and under-resourced and struggle to cope with the cumulative burden of infectious and chronic diseases. An estimated 80% of regional health budgets has been allocated to communicable disease for the last decade [3,4]. Health ministries acknowledge the presence and impact of a chronic disease burden, but few countries have chronic disease plans or policies [5]. Historically, formal healthcare in Africa has developed in response to acute communicable diseases and diseases of environmental degradation and pollution [6]. Therefore most health systems prioritise training and expertise in communicable disease and underestimate the importance of building human and material capacity for chronic disease care. Many hospitals and clinics lack basic equipment for effective diagnosis and treatment, few health workers have specialist chronic disease training and chronic disease knowledge among health workers is poor. In many countries high rates of avoidable complications and deaths have been attributed to weak health systems [7-10]. There is a strong consensus that Africa faces significant challenges in chronic disease research, practice and policy.

This special issue in *Globalization and Health *presents new empirical evidence and comprehensive reviews on the local and global challenges of Africa's chronic disease burden. It is the product of a workshop organised by the UK-Africa Academic Partnership on Chronic Disease at the London School of Economics and Political Science (LSE) in June 2008. The partnership was established in 2006 with funding from the British Academy and managed by lead partners at Cambridge University and the University of Ghana. It now constitutes a network of social and medical scientists working from nine countries in Africa, Asia and Europe and from the US who are collaborating on interdisciplinary models for chronic disease research, intervention and policy to help to address the public health challenges posed by chronic diseases in Africans both in Africa and in the diaspora (see appendix 2). The LSE workshop brought together established and young career researchers from Africa, UK and other European countries to explore chronic disease research, practice and policy for African communities.

Previous issues in other journals have focused either on the global chronic disease burden (e.g *Lancet*, 2005 [11]) or on Africa's disease burden with an emphasis on infectious diseases (*BMJ*, 2005 [12]). *Globalization and Health *has featured articles in the past that discuss the role of globalization and global health governance on chronic disease determinants (e.g Hawkes (2006) on the nutrition transition [13]) or on policy development (e.g Magnussen, 2007 [14]), but with limited focus on Africa. This special issue focuses on Africa's chronic disease burden, offers multidisciplinary analyses of challenges and identifies practical and policy solutions. We group and introduce the eight papers under four categories to reflect the workshop panel themes: (1) Medical dimensions of chronic disease in Africa; (2) Psychological dimensions of chronic disease in Africa; (3) Role of communities in tackling chronic disease; and (4) National and international dimensions of fighting chronic disease.

### Medical dimensions of chronic disease in Africa

Sub-Saharan Africa is the only region of the world in which infectious diseases still outnumber chronic diseases as a cause of death. It is perhaps therefore an understandable misperception that chronic diseases are not an important contributor to the burden of disease. In fact, in adults, overall age specific mortality rates from chronic diseases are higher, often several fold higher in younger adult age groups, than in most high income countries [15]. Even more surprising perhaps is that the available data suggest that age specific mortality rates from chronic diseases as a whole are actually higher in sub Saharan Africa than in virtually all other regions of the world, in both men and women [1]. In addition, there is increasing evidence for adverse interactions between some chronic diseases and infectious diseases.

The first paper in this section, of Agyeman, Addo, Bhopal, de Graft Aikins and Stronks [16], provides a thorough review of cardiovascular disease and its risk factors in African origin populations. It finds that African origin populations outside Africa tend to have high stroke mortality rates, with at least part of the explanation for these being high levels of hypertension and diabetes. Historically, they have also tended to have a relatively favourable lipid profile, with for example, on average higher levels of protective HDL cholesterol and lower levels of triglyceride. This has been part of the explanation for lower levels of coronary heart disease in African compared to European origin populations. In the past, these differences in lipids between African and European origin populations have been considered universal, and interpreted as an example of genetic differences. However, this paper cautions against such generalisations, with examples of migrant African populations where this and other characteristics typically associated with African origin populations are not found. Undoubtedly environmental and behavioural determinants are also very important, and the relative balance of genetic and non-genetic factors in explaining differences between populations of different ancestries remains to be determined.

The paper by BeLue, Okoror, Iwelunmor, Taylor, Degboe, Agyemang and Ogedegbe [17], explores the socio-cultural contexts within which increasing risk of CVD is developing in sub-Saharan Africa. Underlying, upstream, determinants include rapidly increasing urbanisation, poverty and lack of government programmes for the prevention of CVD and related chronic diseases. Some of the well known more proximal behavioural and biological determinants are considered "gendered", with marked differences in their prevalence between men and women. Smoking, for example, tends to be much commoner in men than women, whereas the opposite is true for obesity. A crucial perspective on understanding why some risks are gendered, and indeed on the emergence of CVD risk in general and what can be done to prevent it, is how culture shapes perceptions and experiences of risk and disease. A model, known as PEN-3 [18], is described which provides a framework for examining health beliefs, decisions and behaviours and assist with planning culturally appropriate interventions. Only if CVD risk is understood through the lens of local communities, and is addressed with their involvement, is sustainable change possible. The crucial roles of communities in addressing the chronic disease burden is considered further below, which highlights three other contributions relevant to this area.

Rapidly increasing levels of CVD and diabetes in sub Saharan Africa are occurring alongside continuing high rates of infectious diseases. These two broad types of diseases do not simply exist in parallel, but can actually interact, one exacerbating the other. For example, diabetes increases the risk of developing active tuberculosis (TB), and the presence of diabetes in TB is associated with poorer outcomes. So, increasing levels of diabetes in populations that also have high levels of TB may complicate efforts at successful TB control and treatment. Another example is the potential for anti retroviral therapy (ART) to increase the risk of diabetes and other cardiovascular risk factors, particularly lipid abnormalities. Therefore the successful roll out of ART may need to be linked to efforts to prevent these adverse consequences, and where they do occur to provide appropriate treatment. Young, Critchley, Johnstone and Unwin [2], discuss these two examples and their implications in detail, and help to highlight the urgent need for new research on these areas within sub Saharan Africa.

### Psychological dimensions of chronic disease in Africa

Chronic diseases cause disruptions to the physical capabilities, social identities and life trajectories of sufferers [19]. Studies on experiences of asthma, cancer, diabetes and sickle-cell anaemia in sub-Saharan Africa show that experiences are characterised by depression [20-23], 'chronic unhappiness' [24], spiritual distress [22], 'psychiatric disturbance' [25], and 'suicidal ideation' [22]. These psychological, emotional and spiritual disruptions can occur even within the context of strong family support and often undermine social and medical relationships [2,6] as well as illness management and self-care [20,22,26].

The WHO (2005) observes that "chronic diseases can cause poverty in individuals and families, and draw them into a downward spiral of worsening disease and poverty" (p. 61). The downward spiral has psychological consequences. Poverty may intensify healershopping. A range of medical systems provide chronic disease care in many African countries, including biomedical services, traditional medicine and faith healing systems. Biomedical services are often inaccessible to the poor and may be harmful at point of access due to lack of appropriate chronic disease expertise, medical supplies and equipment. Alternative healers, who are often sought because they may be cheaper and because they offer psychological and spiritual support that is lacking in formal biomedical care, may also be medically harmful for similar reasons. The medical complications that often ensue from healershopping maintain the vicious cycle of physical and psychological disruption [20,26].

Chronic disease management makes demands on the time, emotions and physical capabilities of caregivers. Increasing dependence on family and significant others for medical care and self-care, particularly within contexts of poverty, can cause emotional conflict and breakdown in marital and intimate relationships, as well as family abandonment [20,27]. Finally, stigma is a significant psychological stressor. In many African countries, physical chronic conditions such as diabetes, cancers and epilepsy and mental illnesses like schizophrenia and psychosis are stigmatised [26-28]. The different facets of stigma [29] and their consequences are documented. Actual stigma leads to discrimination and ostracism of people with aforementioned conditions. Courtesy stigma leads to discrimination of caregivers and significant others of the chronically ill. Perceived stigma leads to self-imposed socially restricted lives for both groups.

Four original papers in this special issue contribute to the literature on the psychological impact of chronic disease. They underscore the importance of understanding and tackling this challenge through multi-level interventions that encompass medical, psychosocial, economic and, for stigmatised conditions, rights-based support. Kolling, Winkley and von Deden present new anthropological data on diabetes experiences among the urban poor of Dar es Salaam, Tanzania [30]. They describe the intricate relationship between physical and psychological disruption. They also show how poverty breeds a double burden of disease in households, such that adults living with diabetes must make difficult choices between paying for their own care and sacrificing their health needs for children living with infectious diseases. Anie, Egunjobi and Akinyanju, examine the psychosocial impact of sickle cell disorder in 408 adolescents and adults attending three hospitals in Lagos, Nigeria [31]. Pain and disability are central features of sickle cell disease; sickle cell interventions prioritise pain management and encourage home-based 'active coping skills'. However, this study demonstrates that outcomes on pain management and coping are complex and unpredictable: they are shaped by the interplay between culturally mediated health and sickle cell beliefs, mood, subjective evaluation of self-control and self-efficacy, and the quality and accessibility of healthcare. Read, Adiibokah and Nyame present anthropological work on Ghanaian rural experiences of severe mental illness [32]. These experiences are characterised by poor knowledge and understandings of mental illness, family stress and disruption, actual and courtesy stigma, poor formal mental healthcare and healershopping. The study highlights the way weak health systems and inadequate policies deepen the negative psychosocial impact of chronic mental illness for sufferers and their families, and underscores the need for rights-based interventions. Similarly, Skovdal and Ogutu's social psychological study on Kenyan child carers of adults living with HIV/AIDS highlights the complex psychosocial course of caregiving [33]. Child carers experience daily physical, financial and educational disruptions. But the psychological impact of care-giving varies among carers. Children evaluate their experiences along a continuum of good coping to bad coping depending on their unique circumstances and personalities and access to resources. The authors emphasise that the development of interventions to support child carers must take into account the way Kenyan children actively construct subjectivities and social identities within the context of community health crisis.

### Role of communities in tackling chronic disease

The academic literature on chronic diseases in Africa often emphasises the top-down development of policy and the need to improve and expand medical services. However, most of the burden of care of the chronically ill is carried by patients' families, households and communities - even more so in rural areas which often lie beyond the reach of policies and services.

Furthermore the interaction between well-intentioned policy and health services on the one hand, and patients and their families on the other is seldom a seamless process. Communities play a key role in shaping the lifestyle decisions that drive chronic illness, and peoples' interpretations of and responses to pain and suffering [34]. Community networks play a key role in determining the success of the diffusion of health-related knowledge from health professionals to vulnerable individuals [35]. Community-level norms and practices shape whether or not people will make best use of medical services when these are available, in terms of appropriate access and optimal adherence to medical advice.

Three papers make an important contribution to understandings of the need for community participation in efforts to build health-supporting social environments. De-Graft Aikins, Boynton and Atanga provide a comprehensive overview of the structural, community and individual dimensions of tackling chronic disease in Ghana and Cameroon [36]. They discuss the role of community interventions in bridging the gap between 'social logic' and 'medical logic' regarding health and illness, and in contributing to the vexing challenge of translating abstract health expertise and policy into effective practice in real social settings. They refer to the potential of preventive health interventions by grassroots community and faith-based organisations, and of patient-led self-help and advocacy groups for the chronically ill. They indicate the 'knowledge broker' role of the mass media, and popular community opinion leaders - locating these against the backdrop of wider national and international policy initiatives, and the cultural realities in which individual patients are located.

Read, Adiibokah and Nyame's ethnographic study highlights the immense suffering resulting from the chaining and beating of the chronically mentally ill in Ghana [32]. They demonstrate the chasm between international human rights discourses and Ghanaian human rights legislation on the one hand, and the grim realities of patients and their carers, often in remote and under-served settings, on the other. Individualistic and 'top down' conceptualisations of human rights have little resonance in a context where most mentally ill people have no access to medical treatment or support, and where chaining is often regarded as the only option available to families and healers within a cultural context that prioritises the safety and moral integrity of the group over the individual. Less violent treatment of the mentally ill is unlikely to evolve in the absence of proper community participation, including the bottom-up engagement of affected families, healers and other players involved in methods such as chaining, and the development of viable local-level support for the mentally ill and their families.

Skovdal and Ogutu's study of coping responses by child carers in western Kenya, highlights the limitations of top-down support interventions that fail to take account of how local child carers, their 'patients' and communities make sense of and respond to the challenges of illness and caring [33]. They criticise the knee-jerk tendency of many international agencies to unproblematically assume that children are damaged individuals in need of individual-level counselling. Whilst their study is not blind to the suffering of many child carers, they conceptualise children's coping and resilience in terms of the availability of community-level support and in terms of children's ability to access this support, rather than defining the children in terms of their individual mental health. Rather than prioritising counselling services, efforts to support young carers should focus on providing communities with the financial and social psychological support they need to support child carers appropriately.

These papers, and that of BeLue et al described in the section above on medical dimensions, make a compelling case for community participation as an essential precondition for effective prevention, care and support of the chronically ill. There is an urgent need for health professionals and policy makers to acknowledge this, and also to promote proper community consultation and involvement through formal recognition of the vital role of community outreach skills and activities in the training and job description of medical and welfare personnel. Currently many doctors, nurses and social workers are trained in traditional one-to-one forms of communication with patients and clients [37]. Community outreach needs to be identified as a core training specialisation in all health and welfare related education. Within health services there needs to be formal ring-fencing of time and resources to enable proper community engagement to occur.

### National and international dimensions of fighting chronic disease

International health agencies and national governments are beginning to recognize and confront the significant global burden of chronic diseases. The WHO has been instrumental with its 2005 publication "*Preventing chronic disease: a vital investment*" which proposed that enough is known about the causes, prevention and treatment of the major chronic diseases to inform strong advocacy for changes in priority setting and reallocation of resources towards chronic disease prevention in developing countries [1]. Tobacco control efforts over the last decade in Europe and America, for example, have demonstrated the utility of multi-faceted interventions - legislation, fiscal, and population-based interventions - for chronic disease health protection [38]. The WHO has emphasized their global goal of reducing chronic diseases by 2% every year between 2005 and 2015, thereby preventing 36 million deaths [39]. For most African, Asian and Latin American countries, the 2015 target is off track. Since 2007 the World Bank and a growing number of international agencies have joined the WHO in calling for more resources devoted to chronic disease management [40] and for coordinated effort by national leaders to strengthen chronic disease prevention and control efforts [41-44]. International non-governmental organizations have also increased their commitment to reducing the global burden of chronic diseases by fostering collaborations with partners in the public and private sectors. For example the National Heart, Lung, and Blood Institute (NHLBI), a component of the US National Institutes of Health and UnitedHealth Group, one of the world's largest health and wellbeing companies, have forged a collaboration to counter chronic diseases by supporting a collaborative global network of centres of excellence (COEs) in low-income and middle-income countries [45]. The goal is to support research that will generate evidence to inform policy decisions. Furthermore, a campaign of 'international science advocacy' led by the Chronic Disease Action Group - a collaboration between *The Lancet *and scientists from WHO and a wide range of countries - has contributed to the development of international health strategies since 2007 [44]. In an enlightening editorial on "Chronic diseases and calls to action" Shah Ebrahim (2008) noted that the real challenge for any call to action is to develop and implement a plan for achieving its goals [46]. Experts endorse plans and policies that simultaneously address structural (including policy, fiscal, industry and private businesses, international collaboration), community (including mass media, voluntary organization, institutions, primary healthcare) and individual (including behavioural and pharmacological interventions) dimensions of chronic diseases [1,10].

South Africa, Mauritius, Tanzania and Cameroon are among the few African countries that have responded to the call for action [34]. However these countries have focused on limited aspects of the ideal policy structure. In the majority of countries, there is a gap between policy makers' recognition of a national chronic disease burden and the development and implementation of chronic disease policies and plans.

A major problem is a lack of political will. In this issue, de-Graft Aikins et al [34] highlight some factors that maintain a lack of political will in Ghana, despite the fact that some chronic diseases have been placed on the health ministry's priority intervention lists since the early 1990s and a grassroots movement for chronic disease advocacy exists. The factors include a fragmented chronic disease research community with limited power to influence policy and policymakers' overdependence on international health directives and funding in the development of local policies. The authors observe that other countries with similar chronic disease profiles to Ghana are likely to face similar policy and political challenges.

A second problem is a weak culture of knowledge transfer. While African production of chronic disease research is extremely low compared to the rest of the world, multidisciplinary research does exist on the epidemiological, medical, socio-cultural and psychological dimensions of chronic diseases in the region. This limited body of work provides important insights for practice and policy. The challenge is in undertaking the sorts of analyses that enable appropriate knowledge transfer and knowledge exchange based on extant research.

Reviews such as the special issue papers by Agyeman et al [16] on the risk factors for chronic diseases among the African diaspora in Europe, by Belue et al [17] on the cultural dimensions of the CVD burden, and by Young et al [2] on the co-morbidity between infectious and chronic diseases are a critical contribution towards an analysis of knowledge transfer. Agyeman et al [16] establish in their review the risk factors for, and higher prevalence of, hypertension and diabetes in migrant African populations in Europe compared to the host populations of the countries of resettlement and populations in their home countries. The implications are clear to the planning of services for these minority groups within the context of their European host countries. The review also offers insights on socio-cultural continuities and change within migrant populations (e.g the area of dietary practices and physical activity), which provide some lessons for African policymakers. The paper emphasises the importance of conducting comprehensive reviews of the epidemiology of major chronic diseases in Africa to provide more concrete, context specific evidence for policy.

Belue et al [17] provide an important perspective by focusing on the cultural dimensions of CVD prevention, control and treatment across the continent. In general discussions of the burden of chronic disease in non-western contexts, culture is either ignored or is treated merely as a barrier to medical adherence or good self-care. Yet as some empirical papers in this issue suggest, fighting the chronic disease burden in Africa requires critical attention to the way society and culture structure lay concepts of health, disease and specific chronic diseases, how practical and existential responses to chronic disease experiences develop and are sustained, and how social support and alternative healthcare systems are shaped by culture, religion and contemporary economic forces. Policymakers have to consider the extent to which the language, content and objectives of expert information and health services reflect these socio-cultural contexts. Data and insights from anthropological and psychological research are therefore crucial to the development of socio-culturally appropriate chronic disease policies.

The application of the knowledge of the pathophysiology in co-morbidities of chronic diseases with chronic infectious diseases such as HIV and TB is evident. Young et al [2] review the evidence on the limited but compelling data on the epidemiology of co-morbidities and the likely effects of drug interactions. Knowledge transfer here would need to take into account the need to strengthen the capacity of African health systems to address health financing in this area, to improve procurement and access to medicines and to introduce multi-disciplinary working practices across areas of human resource specialisation for medical and social care. Although the literature from the social sciences was not included in the review, the authors touch on the potential effects of factors such as gender and poverty which would add significant socio-cultural challenges to interventions in this area.

## Conclusion

The evidence presented in this special issue on the medical, psychological, community and policy dimensions of the chronic disease burden in sub-Saharan Africa emphasises the urgent need for primary and secondary interventions and for African health policymakers and governments to prioritise the development and implementation of chronic disease policies. In addition, the contributions in this issue help to identify gaps in the evidence that is needed to guide the development and implementation of preventive interventions and policies. These gaps fall in two broad categories. One category concerns the need for multidisciplinary models of research, bringing together a range of perspectives, including cultural, psychological, epidemiological, clinical and economic, to properly inform the design of interventions that address the needs of communities and individuals at risk of chronic diseases and their consequences. The second broad category of research, complementary to the first, concerns understanding the processes and political economies of policy making in sub Saharan Africa. This is essential to be able to effectively influence the development and implementation of policies supporting the prevention and care of chronic diseases. Undoubtedly this will include gaining a better understanding of the economic impact of chronic diseases within sub Saharan Africa. It will also include understanding better the relationships between national policy making and international economic and political pressures, which have a huge impact on the risk of chronic diseases and the ability of countries to respond to them.

## Appendices

### Appendix 1

Chronic diseases are often referred to as 'non communicable diseases' to distinguish them from communicable diseases and as 'diseases of lifestyle' to distinguish them from diseases with environmental causes. These terms ignore the similarities between the categories: some non communicable diseases have infectious elements; diseases of lifestyle also have causal elements in the environment. In this paper we adopt the WHO (2005) definition of 'chronic diseases' which refers to a set of conditions with "important shared features" including: (1) "chronic disease epidemics take decades to become fully established - they have their origins at young ages"; and (2) given their long duration, there are many opportunities for prevention; and (3) they require a long-term and systematic approach to treatment" (p.35)

### Appendix 2

Visit http://www.psych.lse.ac.uk/chronicdiseaseafrica or click Africa-UK Partnership at LSE Health http://www2.lse.ac.uk/LSEHealthAndSocialCare/LSEHealth/Home.aspx for more details on the UK-Africa Academic Partnership on Chronic Disease.
